# Editorial: Big data and artificial intelligence technologies for smart forestry

**DOI:** 10.3389/fpls.2023.1149740

**Published:** 2023-02-10

**Authors:** Weipeng Jing, Zhufang Kuang, Rafał Scherer, Marcin Woźniak

**Affiliations:** ^1^ School of Information and Computer Engineering, Northeast Forestry University, Harbin, China; ^2^ Central South University Forestry and Technology, Changsha, China; ^3^ Department of Intelligent Computer Systems, Częstochowa University of Technology, Częstochowa, Poland; ^4^ Faculty of Applied Mathematics, Silesian Unversity of Technology, Gliwice, Poland

**Keywords:** smart forestry, artificial intelligence, neural networks, IoT - internet of things, big data & analytics

Machine learning and data analysis are becoming increasingly important in the field of Smart Forestry, as it allows for the analysis of large amounts of data in order to make predictions and identify patterns. This can be used for a variety of purposes, such as predicting the growth and health of trees, identifying areas that are at risk of disease or pests, and optimizing the management of forests. Additionally, machine learning can be used to analyze satellite and drone imagery, which can provide valuable information about the condition of forests and help with monitoring and conservation efforts.

Overall, machine learning enables Smart Forestry to be more efficient, effective, and sustainable. There are various types of data that can be analyzed in the context of Smart Forestry using machine learning. Some examples include:

1. Climate data: Information about temperature, precipitation, and other weather conditions can be used to predict the growth and health of trees, as well as identify areas that may be at risk of disease or pests.
2. Soil data: Data about the chemical and physical properties of soil, such as pH levels, can be used to predict the growth and health of trees, and to identify areas that may be suitable for different types of trees or forestry practices.
3. Remote sensing data: Satellite and drone imagery can provide valuable information about the condition of forests, such as tree cover, canopy height, and biomass. This can be used to monitor changes in forests over time and to identify areas that may be at risk of deforestation or degradation.
4. Inventory data: Information about the number, species, and size of trees can be used to predict future growth and health of the forest, and to optimize the management of the forest.
5. Harvest data: Information about past harvesting practices can be used to optimize future harvesting schedules and methods.

Overall, machine learning and big data analysis can analyze and make predictions from the large amounts of data generated from various sources, such as sensors, drones, and satellites, to support better decision making in Smart Forestry.

Forest monitoring based on SAR, Lidar, optical remote sensing, and IoT can provide support for large spatial scale forest management and decision-making. With the development of big data technologies, the speed of smart forestry construction and the level of forestry information management has significantly improved. On the one hand, high-performance architectures for big data can significantly improve the efficiency of large-scale forestry research; on the other hand, artificial intelligence models can effectively extract the vegetation features and ecological parameters of the forest from the remote sensing data. Therefore, the development, integration, and application of big data technology have become the focus of forestry research. Meanwhile, research on forest plants driven by data and process-based models has also received much attention. Special attention is paid to the application of smart forestry based on big data and remote sensing.

This Research Topic entitled “*Big Data and Artificial Intelligence Technologies for Smart Forestry*” is a collection of papers dealing with forest monitoring and analysis and presents the scientific research achievements of emerging technologies such as big data, remote sensing and IoT applied in the field of forestry. We are pleased to present a collection of accepted papers which present interesting ideas and results of experiments that contribute to the field of smart forestry by the use of artificial intelligence models.

## Author contributions

All authors listed have made a substantial, direct, and intellectual contribution to the work and approved it for publication.

